# Fish oil-derived lipid emulsion induces RIP1-dependent and caspase 8-licensed necroptosis in IEC-6 cells through overproduction of reactive oxygen species

**DOI:** 10.1186/s12944-018-0786-5

**Published:** 2018-06-23

**Authors:** Jun-Kai Yan, Wei-Hui Yan, Wei Cai

**Affiliations:** 1 Department of Pediatric Surgery, Xin Hua Hospital affiliated to Shanghai Jiao Tong University School of Medicine, Shanghai, China; 2 Shanghai Key Laboratory of Pediatric Gastroenterology and Nutrition, Shanghai Institute for Pediatric Research, Xin Hua Hospital Affiliated to Shanghai Jiao Tong University School of Medicine, 1665 Kongjiang Rd, Shanghai, 200092 China

**Keywords:** Fish oil-derived lipid emulsion, Parenteral nutrition, Necroptosis, IEC-6, Receptor-interacting protein 1, Caspase 8, Reactive oxygen species

## Abstract

**Background:**

Excessive cell death of enterocytes has been demonstrated to be partially associated with the intravenously-administrated lipid emulsions (LEs) during parenteral nutrition (PN) support. However, as a new generation of LE, the effect of fish oil-derived lipid emulsion (FOLE) on the death of enterocytes remains elusive.

**Methods:**

Intestinal epithelial cells (IEC-6 cell line) were treated with FOLE (0.25–1%) for 24 h. Cell survival was measured by CCK-8 assay, and morphological changes were monitored by time-lapse live cell imaging. The expression of receptor-interacting protein 1/3 (RIP1/3) and caspase 8 was assessed by westernblot, and the formation of necrosome (characterized by the assembly of RIP1/3 complex along with the dissociation of caspase 8) was examined by immunoprecipitation**.** Additionally, the production of intracellular reactive oxygen species (ROS) was detected by using a ROS detection kit with an oxidation-sensitive probe (DCFH-DA).

**Results:**

FOLE dose-dependently induced non-apoptotic, but programmed necroctic cell death (necroptosis) within 4–8 h after treatment. The assembly of RIP1/3 complex along with the dissociation of caspase 8 from RIP1 was observed in FOLE-treated cells. Moreover, FOLE-induced cell death was significantly alleviated by inhibiting RIP1, and was further aggravated by inhibiting caspase 8. In addition, prior to cell death the accumulation of intracellular ROS was significantly increased in FOLE-treated cells (increased by approximately 5-fold versus control, *p* < 0.001), which could be attenuated by inhibiting RIP1 (decreased by approximately 35% versus FOLE, *p* < 0.05).

**Conclusions:**

FOLE induces RIP1-dependent and caspase 8-licensed necroptosis through overproduction of ROS in vitro. Our findings may provide novel insights into the clinical applications of FOLE during PN support.

**Electronic supplementary material:**

The online version of this article (10.1186/s12944-018-0786-5) contains supplementary material, which is available to authorized users.

## Background

Excessive cell death in enterocytes is a great challenge for the administration of parenteral nutrition (PN), which may lead to intestinal atrophy, loss of epithelial barrier function, and even parenteral nutrition associated liver disease (PNALD) [1, 2]. Currently, several factors that may affect the homeostasis of intestinal epithelium during PN have been studied, including inflammatory cytokines [3, 4], hormones [5, 6], supplementation of enteral nutrition [7] and changes in microbiota [8]. Additionally, a rodent study has recently demonstrated that intravenous lipid emulsion (LE) which serves as one of the key regiments in PN prescription is also involved in the modulation of intestinal homeostasis [9]. As distinct LEs may elicit distinct impacts on enterocytes, this may have significant implications suggesting that the role of LEs is not only a lipid source for energy supply, but also an important modulator of intestinal homeostasis during PN.

Currently, the commercially available LEs for clinical use with various composition of fatty acids include: soybean oil-derived lipid emulsion (SOLE), fish oil-derived lipid emulsion (FOLE) and 80% olive oil-supplemented lipid emulsion (OOLE), among which FOLE is a new generation of LE (the 4th generation) that provides a large content of eicosapentaenoic acid (EPA) and docosahexaenoic acid (DHA), allowing a lower ω-6/ω-3 (1:8) ratio as a significant departure from the LEs of the previous generations [10]. Omegaven® (Fresenius Kabi, Germany) is the only commercially available product available in Canada, Europe and Asia marketed as 100% fish oil. However, though it has been regarded as a therapeutic lipid to ameliorate liver injury [11], the effect of FOLE on the intestinal epithelium remains largely unknown, since both in vivo and in vitro studies designed to address this issue are extremely limited currently. In addition, FOLE is significantly more costly than other LEs, therefore greater discussion is needed to better understand the possible advantages and shortages of this new generation LE product. Recently, increasing evidence has demonstrated that gut-derived cell lines can serve as appropriate models to study the role of PN formula or LEs in vitro [12–16]. Thus, this study was designed to address the effect of FOLE on the death of enterocytes by using rat gut-derived IEC-6 cells as a model in vitro.

Necroptosis is a new type of programmed cell death which shares with necrosis the fact that dying cells display the morphological features of necrosis instead of apoptosis, but is highly regulated by an intracellular protein platform [17]. Herein, we report a significant pro-necroptosis effect of FOLE on IEC-6 cells, which requires receptor-interacting protein 1 (RIP1) and is licensed by caspase 8.

## Methods

### Cell culture and reagents

IEC-6 cells (Cell Bank of the Chinese Academy of Sciences, China) were maintained at 37 °C and 5% CO_2_ in DMEM supplemented with 10% fetal bovine serum. The cell culture reagents were obtained from Life Technologies (Carlsbad, CA, USA). Fetal bovine serum was obtained from MP Biomedicals LLC (Solon, Ohio, USA). Lipid emulsions were derived from commercial products as follows: SOLE (Lipofundin LCT/MCT, 20%, Baxter Healthcare, China), OOLE (Clinoleic, 20%, Baxter Healthcare, China), FOLE (Omegaven, 10%, Fresenius Kabi, China). Mouse anti-RIP1, caspase 8 and GAPDH, and rabbit anti-RIP3 were purchased from Santa Cruz (Santa Cruz, CA, USA). Z-IETD-FMK was purchased from Santa Cruz (Santa Cruz, CA, USA). Necrostatin-1 (Nec-1), necrosulfonamide (NSA) and all other chemicals were purchased from Sigma (St. Louis., MO, USA).

### Cell treatment

IEC-6 cells were exposed to 0.25–1% FOLE for 1–24 h to test the effect of FOLE on the viability of IEC-6 cells. In some experiments, cells were pre-treated with the following chemicals: Z-IETD-FMK (20 μM), Nec-1 (20 μM) and NSA (10 μM).

### Cell viability assay

Cell viability was evaluated using the Cell Counting Kit-8 (CCK-8) assay kit (Dojindo, Japan). Approximately 5 × 10^3^ cells per well were cultured in 96-well culture plates for 24 h. After FOLE treatment, 10 μl CCK-8 was added to each well and incubated at 37 °Cfor 30 min. Absorbance was measured using Multiskan Spectrum (Thermo) at 450 nm.

### Measurement of caspase 8 activation

Caspase 8 activity was measured using a Caspase 8 Activity Kit (Beyotime CII51, China) according to the manufacturer’s instructions. Briefly, cells were washed with cold PBS, resuspended in lysis buffer on ice for 15 min. The lysate was centrifuged at 16,000 g at 4 °C for 15 min. The activity of caspase 8 was measured using substrate peptides Ac-IETD-pNA. The release of p-nitroanilide (pNA) was quantified by determining the absorbance with Multiskan Spectrum (Thermo) at 405 nm.

### Immunoprecipitation assay

Immunoprecipitation was performed using an immunoprecipitation kit (Thermo, Rockford, USA) according to the manufacturer’s instructions. In brief, RIP1 antibody was coupled to the AminoLink Resin in a Spin Column and stored at 4 °C. Cell lysate was incubated with the prepared anti-RIP1-coupled resin at 4 °C overnight. After incubation, excess proteins were removed, and the target proteins bound to the anti-RIP1-coupled resin were eluted for western blotting analysis.

### Western blot analysis

Cells were harvested and lysed on ice for 30 min in 100 μl of RIPA buffer. The protein concentration was determined using a BCA protein assay kit (Thermo, Rockford, USA). Aliquots of the lysates (30 μg of protein) were loaded onto a NuPAGE Bis/Tris gel (Thermo, Rockford, USA), followed by transferring to PVDF membranes using an iBlot® Dry Blotting System (Thermo, Rockford, USA). After blocking in 5% BSA for 30 min at room temperature, the membranes were incubated with the indicated primary antibodies at 4 °C overnight. The immunoblots were detected with a ChemiDoc XRS+ system (Bio-Rad, Hercules, CA, USA).

### Immunofluorescence staining

Immunofluorescence staining was performed according to standard procedures. Briefly, cells were fixed in 3.7% formaldehyde at room temperature for 15 min, and then permeabilized with 0.2% Triton-X100. After blocking in 5% BSA for 60 min, the cells were incubated overnight at 4 °C with primary antibody recognizing RIP1 or RIP3, and then incubated with secondary antibody conjugated with Alexa Fluor 594 or Alexa Fluor 488 for 1 h at room temperature. Images were visualized using Leica DMI6000B fluorescence microscopy with LAS AF LITE image processing software (Leica, Germany).

### Time-lapse live cell imaging

Confluent IEC-6 cells were cultured in a 6-well culture plate. Immediately after FOLE treatment (0.5%), the culture plate was transferred to an automated stage equipped with a cell incubator that maintains a constant humid environment of 37 °C and 5% CO_2_. Cells were imaged on an inverted microscope (Leica DMI6000B, Germany) and the images were acquired every 1 h for 24 h. Image acquisition was controlled by LAS AF LITE image processing software (Leica, Germany).

### Detection of intracellular reactive oxygen species (ROS)

Intracellular ROS was detected using a ROS detection kit (Beyotime, China). Briefly, cells were incubated with an oxidation-sensitive probe (DCFH-DA, 10 μM) at 37 °Cfor 20 min according to the manufacturer’s instructions. The cells were then washed three times with PBS, and the fluorescence signal was determined by fluorescence microscopy and fluorescence spectrophotometer at 485 nm for excitation and 530 nm for emission. ROSUP (a cocktail of oxidants) served as positive control.

### Statistical analysis

All experiments were performed at least three times, and the results were presented as mean ± standard deviation (SD). Comparisons between two groups were assessed using a Student’s two-tailed t-test, and comparisons between multiple groups were assessed using a one-way analysis of variance followed by the Newman-Keuls post hoc test. A *p*-value < 0.05 was considered to be statistically significant.

## Results

### FOLE induces necrotic-like cell death in IEC-6 cells

**Fig. 1:**
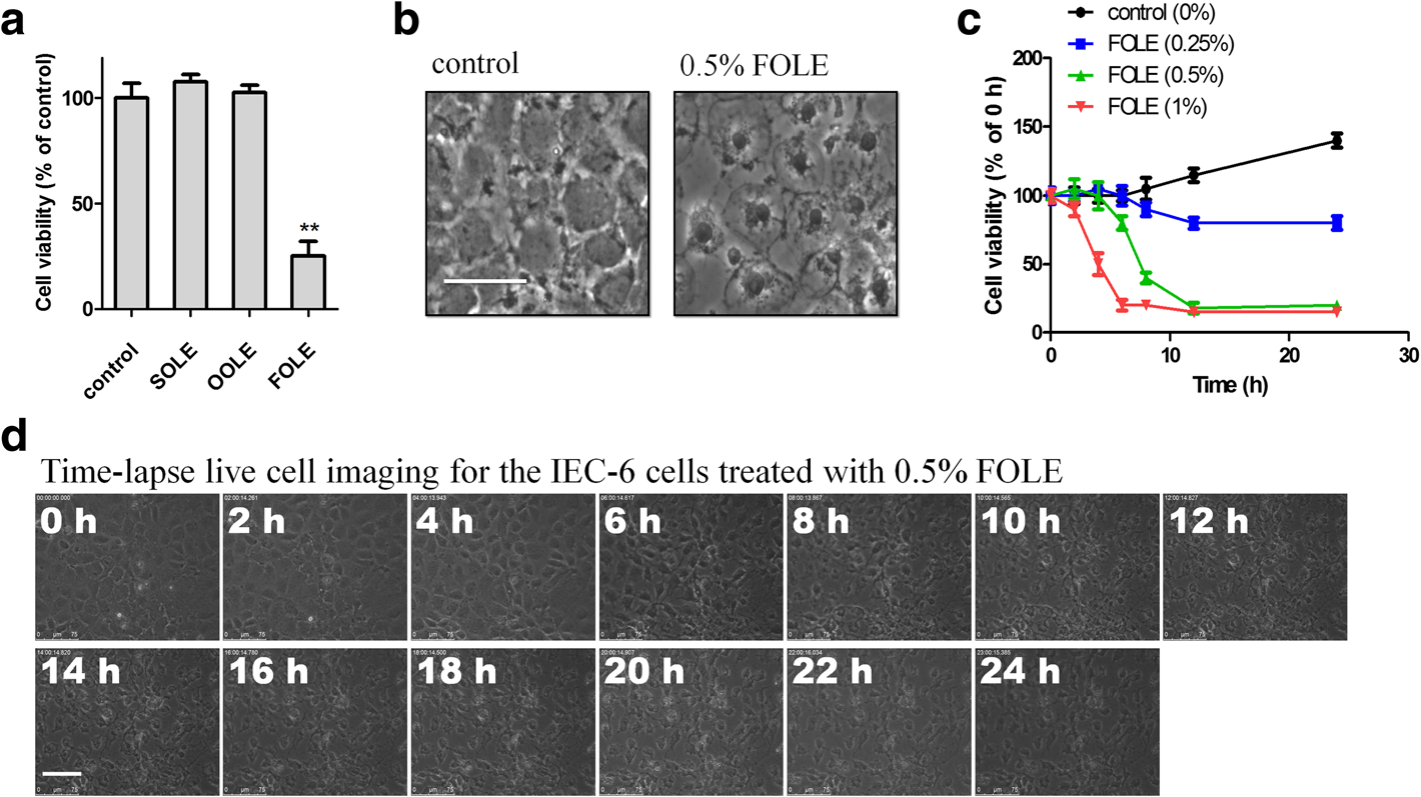
FOLE induces necrotic cell death in IEC-6 cells. **a** Cell viability assessed by CCK-8 assay. IEC-6 cells were treated with 0.25% SOLE, 0.25% OOLE and 0.5% FOLE for 24 h. Note that a significant reduction in the viability was observed in FOLE-treated cells. Data are presented as mean ± SD. **, *p* < 0.01 compared to control (*n* = 6–8 wells/group). **b** Necrotic cell death in FOLE-treated cells. Representative images are shown (Scale bar = 25 μm). **c** Cell viability assessed by CCK-8 assay. IEC-6 cells were treated with 0.25–1% FOLE for 0–24 h. Notably, FOLE reduced the cell viability of IEC-6 cells in a dose-dependent manner, and rapid reduction was observed at 4–8 h post FOLE treatment. **d** Time-lapse live cell imaging. Post-confluent IEC-6 cells were treated with 0.5% FOLE, and a 24-h time-lapse live cell imaging was performed as described in material and methods. Representative images are shown. Scale bar = 75 μm. Videos are available as supplementary data (see Additional file 1: IEC-6 cells treated with FOLE.avi). Three experiments were performed that showed similar results

As Omegaven-derived FOLE contains half the amount of fatty acids in Lipofundin-derived SOLE and Clinoleic-derived OOLE, the initial concentration of FOLE (0.5%) was set at a rate twice as that of SOLE and OOLE treatment (0.25%). As indicated by CCK-8, only FOLE induced a significant reduction in the cell viability at 24 h post treatment, while no significant alterations were observed in the cells treated with SOLE or OOLE (Fig. 1a). Morphologically, non-apoptotic, but necrotic-like cell death was observed in FOLE-treated cells (Fig. 1b).Time course study further revealed that rapid reduction in the cell viability occurred at 4–8 h post FOLE treatment (Fig. 1c), which is consistent with time-lapse live cell imaging showing rapid morphological changes observed at 6 h post treatment (Fig. 1d). Taken together, these results suggested that FOLE induces rapid necrotic-like cell death in IEC-6 cells, and changes during 4–8 h post treatment would be a key for further investigations.

### FOLE-induced necrotic cell death is dependent on RIP1

**Fig. 2:**
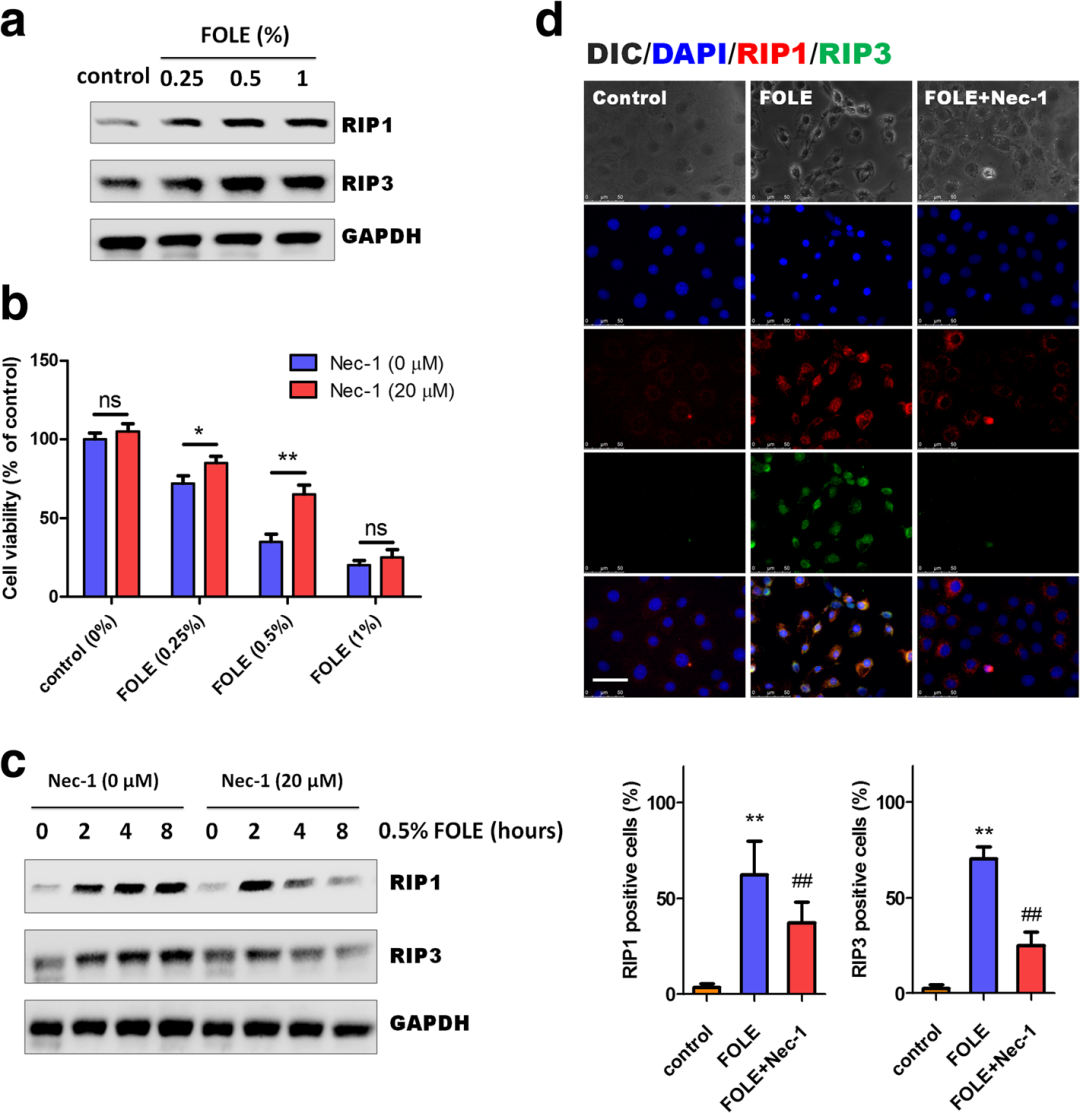
FOLE-induced necrotic cell death is dependent on RIP1. **a** Representative immunoblots of RIP1 and RIP3. IEC-6 cells were treated with FOLE at 0.25–1%, and the indicated proteins were analyzed at 8 h after FOLE treatment. **b** Cell viability. IEC-6 cells were pre-treated with Nec-1 (20 μM) for 40 min, followed by exposure to 0.25–1% FOLE as described in material and methods. The viability was analyzed 8 h after treatment. Data are presented as mean ± SD. *, *p* < 0.05, **, *p* < 0.01 (*n* = 6–8 wells/group). **c** Representative immunoblots of RIP1 and RIP3. IEC-6 cells were pre-treated with Nec-1 (20 μM) for 40 min, followed by exposure to 0.5% FOLE. The indicated proteins were analyzed at 2–8 h after treatment. **d** Representative images of RIP1 and RIP3 staining in IEC-6 cells. Cells were treated as described in (**c**). Scale bar = 50 μm. Three experiments were performed that showed similar results

As shown, 6 h after FOLE treatment, both RIP1 and RIP3 were significantly increased by FOLE treatment in a dose-dependent manner (Fig. 2a). Pre-treatment with Nec-1 (a specific inhibitor for RIP1) significantly attenuated FOLE-induced necrosis at low doses (0.25 and 0.5%); however, no significant effects were observed on the cells treated with 1% FOLE (Fig. 2b). Consistently, western blot analysis demonstrated that FOLE-induced up-regulation of RIP1/3 was evidently suppressed by pre-treatment with Nec-1 (Fig. 2c). Immunofluorescence staining for RIP1 and RIP3 clearly indicated that FOLE significantly induced the expression of RIP1 (red) and RIP3 (green) after 6 h of treatment, which was obviously suppressed by pre-treatment of Nec-1 (Fig. 2d). Collectively, these results suggested that increased expression of RIP1 is required for FOLE-induced cell death.

### FOLE-induced cell death requires the assembly of RIP1/3 complex and is licensed by caspase 8 (Identified as necroptosis)

**Fig. 3:**
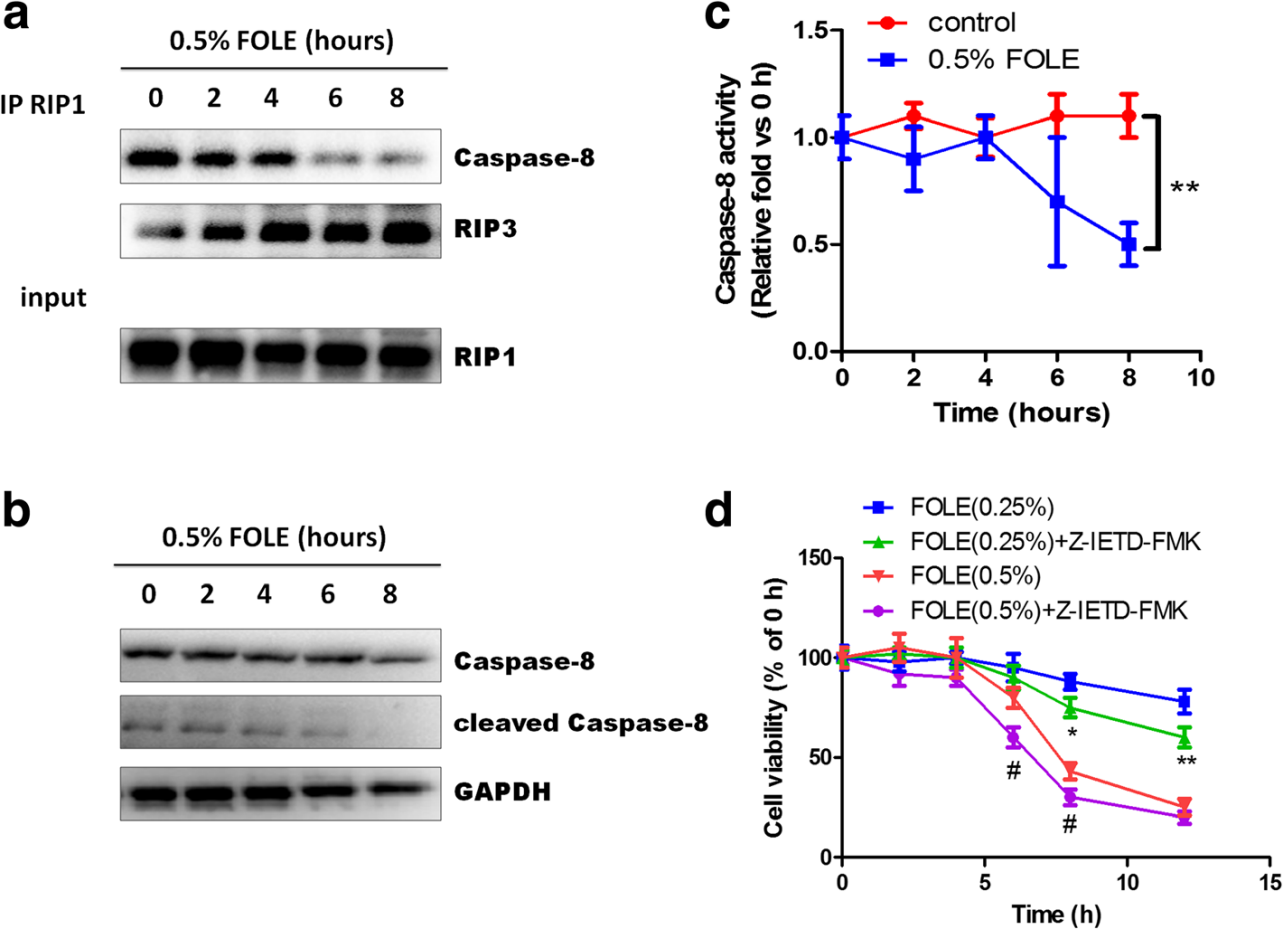
FOLE-induced cell death requires RIP1/3 complex and is licensed by caspase 8. **a** Assembly of RIP1/RIP3 complex and dissociation of caspase 8. IEC-6 cells were treated with 0.5% FOLE for 2–8 h. Whole cell extract was immunoprecipitated with anti-RIP1, and the immunoprecipitates were then immunoblotted with anti-RIP3 or caspase 8. **b** Representative immunoblots of caspase 8. The expression of caspase 8 was analyzed in the cells described above. **c** Caspase 8 activity. Cells were treated as described above. Data are presented as mean ± SD. **, *p* < 0.01 compared to control (*n* = 5–6 wells/group). **d** Cell viability. IEC-6 cells were pre-treated with Z-IETD-FMK (20 μM) for 40 min, followed by exposure to 0.25–0.5% FOLE for 2–12 h. Data are presented as mean ± SD (*n* = 6–8 wells/group). *, *p* < 0.05, **, *p* < 0.01 compared to FOLE (0.25%); #, *p* < 0.05 compared to FOLE (0.5%)

As shown, the formation of necrosome, which is characterized by the assembly of RIP1/3 complex along with the dissociation of caspase 8 from RIP1, was observed in FOLE-treated cells. Interestingly, however, assembly of RIP1/3 complex was already evident at 2 h post treatment, while obvious dissociation of caspase 8 from RIP1 was evident after 6 h of treatment (Fig. 3a). Western blot analysis further revealed that cleaved caspase 8 was time-dependently down-regulated by FOLE treatment, though the total amount of caspase 8 was unchanged (Fig. 3b). Consistently, though no significant alterations were observed within 0–4 h post treatment, the catalytic activity of caspase 8 was significantly decreased at 6–8 h post FOLE treatment (Fig. 3c). Collectively, these results indicated that the formation of necrosome as a marker of necroptosis was observed in FOLE-treated cells. Additionally, inhibition of caspase 8 activity by Z-ITED-FMK further aggravated FOLE-induced cell death, suggesting that FOLE-induced cell death is not only companied with the changes in caspase 8 activity, but also attributed to the loss of caspase 8 activity (Fig. 3d). Give that loss of caspase 8 activity appears immediately prior to the execution of necrotic cell death, we suppose that FOLE-induced necrosis could be identified as programmed necroptosis that requires the assembly of RIP1/3 complex and is licensed by caspase 8.

### FOLE-induced necroptosis is attributed to ROS overproduction instead of MLKL

**Fig. 4:**
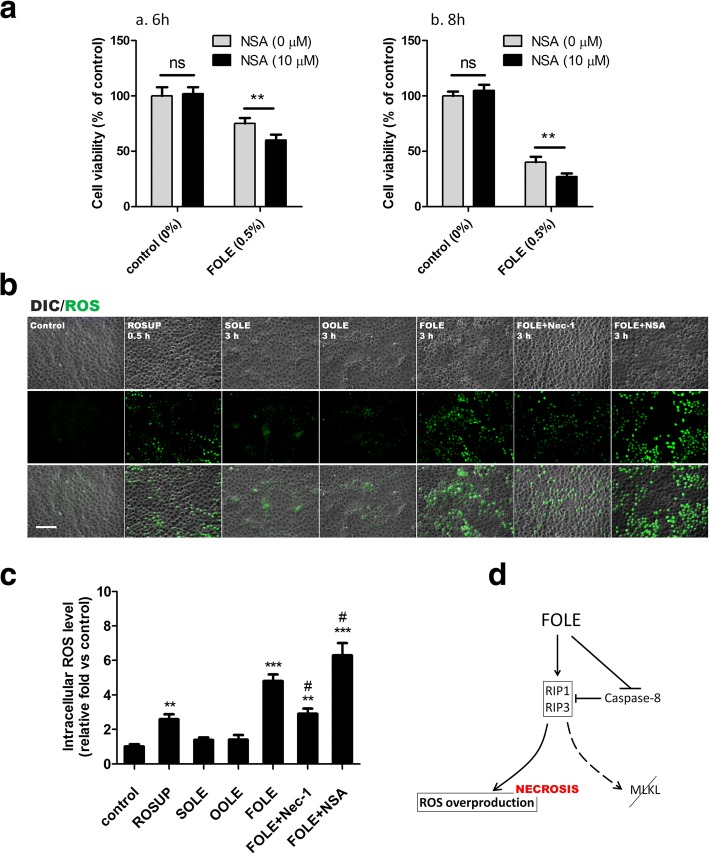
FOLE-induced necroptosis is mediated via ROS overproduction instead of MLKL. **a** Cell viability. IEC-6 cells were pre-treated with NSA (10 μM) for 40 min, followed by exposure to 0.5% FOLE for 6 h (a) and 8 h (b). Data are presented as mean ± SD. **, *p* < 0.01 (*n* = 6–8 wells/group). **b** Representative images of ROS production in FOLE-treated cells. Three hours after FOLE-treatment, the levels of intracellular ROS were determined by DCFDA as described in material and methods. Pretreatment with Nec-1 and NSA was performed as described previously. Treatment with ROSUP (30 min) served as positive control. ROS signal is presented as green fluorescence. Scale bar = 100 μm. Three experiments were performed that showed similar results. **c** Quantification of intracellular ROS levels. Cells were treated as described in (**b**). Data are presented as mean ± SD **, *p* < 0.01 ***, *p* < 0.001 compared to control; #, *p* < 0.05 compared to FOLE (*n* = 6–8 wells/group). **d** A schematic representation showing that ROS overproduction instead of MLKL is at least in part responsible for FOLE-induced necroptosis

As a key RIP3 downstream component, we examined whether mixed-lineage kinase domain-like (MLKL) is involved in FOLE-induced necroptosis. Unexpectedly, however, reduced cell viability was not recovered, but was further aggravated by inhibition of MLKL using necrosulfonamide (NSA, a specific MLKL inhibitor), suggesting that a MLKL-independent pathway may exist responsible for FOLE-induced necroptosis (Fig. 4a). Instead, intracellular ROS was determined because ROS overproduction has been reported to be an alternative pathway responsible for RIP3-mediated necrosis in some cell systems [18, 19]. As shown in Fig. 4b, only weak fluorescence was detectable in SOLE or OOLE-treated cells while strong fluorescence was detected in FOLE-treated cells, suggesting that ROS overproduction is a key feature of FOLE treatment distinct from SOLE and OOLE. Importantly, pretreatment with Nec-1 reduced ROS production, while pretreatment with NSA further enhanced ROS production. The relative folds quantified using a fluorescence spectrophotometer further revealed that FOLE-induced ROS production was reduced by approximately 35% with Nec-1 treatment (*p* < 0.05), and was enhanced by approximately 24% with NSA treatment (*p* < 0.05) (Fig. 4c). A schematic representation is shown: FOLE induces necroptosis in IEC-6 cells partially through ROS overproduction instead of MLKL (Fig. 4d).

## Discussion

Intravenous LEs may have differing biological and clinical consequences, since they are unique in the fatty acid profile and additive content. It is between 1920 and 1960 that scientists in both the United States and Japan developed hundreds of LEs with varying compositions [20]. However, though there has been almost one hundred years since the first intravenous LE developed in the United States (Lipomul, produced by Upjohn Co. Kalamazoo, MI, USA), one area that has not been fully studied is the effect that LE products might have on the modulation of intestinal epithelium. A rodent study that compared three commercial LEs in a rat model of PN, including SOLE, OOLE and SMOF (a combination of soybean oil, medium-chain triglycerides, olive oil and fish oil emulsion), has recently suggested that distinct LEs may elicit distinct effects on the intestinal homeostasis [9]. Additionally, an in vitro study using Caco-2 cells demonstrated that OOLE, but not SOLE or FOLE, specifically induces the apoptosis via CELF1/AIF pathway [14]. However, known as a new generation of LE, the effect of FOLE on the intestinal epithelium still remains unclear. Therefore, this study was designed to address this issue by using rat gut-derived IEC-6 cells as a model in vitro.

Striking differences were noted in this study when three LEs were examined. Though no significant alterations were observed on SOLE or OOLE treatment, evident reduction in the cell viability was observed by 24-h FOLE treatment (Fig. 1a). Similar results were obtained when colorectal cancer cells were used as an in vitro model previously elsewhere. Alexi et al... reported a significant reduction in the viability of HT-29 cells after 72 h exposure to 0.7% FOLE (7.06 ml lipid emulsion/l medium), and importantly, however, this reduction could not be attributed to apoptosis [21]. In this study, we noticed that rapid reduction by FOLE appears at 4–8 h after treatment, suggesting that a more aggressive death type may exist instead of apoptosis (Fig. 1c). Morphological analysis by time-lapse live cell imaging showed no signs of apoptosis, but rather, necrotic cell death with swelling of the cell membrane (Fig. 1d). Interestingly, these results obtained from IEC-6 cells were consistent with Harvey et al’s study, in which human aortic endothelial cells (HAECs) presented similar responses to FOLE treatment, including significantly decreased cell viability due to necrotic cell death [22].

As known, necroptosis or regulated necrosis has been recently described as a novel cell death pathway, morphologically similar to that of necrosis, but occurring in a programmed manner through well-defined biochemical pathways. Typically, it is initiated by interaction between RIP1 and RIP3, which originate in the necrosome. At the functional level, the auto- and trans-phosphorylation of RIP1 and RIP3 promote the assembly of necrosome and activation of necroptotic signaling transduction [23]. In this study, we found that FOLE-induced necroptosis was mediated via RIP1/3 complex, as Nec-1 (a specific inhibitor of RIP1) markedly suppressed the expression and association of RIP1/3, and partially alleviated FOLE-induced reduction in cell viability at 0.25 and 0.5% concentration (Fig. 2). However, as no effect of Nec-1 on 1% FOLE treatment, we assume that RIP1-dependent necroptosis and RIP1-independent necrosis may both exist in response to FOLE treatment, and necroptosis may be converted to necrosis with the increasing concentration of FOLE treatment. In addition to the assembly of RIP1/3, we also found that disassociation of caspase 8 is required for FOLE-induced necroptosis. Caspase 8 is a cysteine protease critically involved in regulating cellular apoptosis, and it has recently been shown to suppress RIP1/3-dependent necroptosis [24, 25]. On the molecular level, it has been demonstrated that caspase 8 proteolytically cleave and inactivate RIP1 and RIP3, thereby regulating the initiation of necroptosis [26]. In this study, our results indicated that the activity of caspase 8 was significantly decreased at 6–8 h post FOLE treatment, immediately followed by the initiation of morphological changes of necroptosis. Moreover, inactivation of caspase 8 by its specific inhibitor (Z-IETD-FMK) significantly further decreased the cell viability in comparison to FOLE treatment alone (Fig. 3), suggesting that FOLE-induced necroptosis not only requires the assembly of RIP1/3, but is also licensed by caspase 8. However, one limitation of this study is that the precise mechanism by which FOLE inhibited caspase 8 activity and induced the dissociation of caspase 8 from RIP1 remains unknown, and thus it would be of great importance to address this issue in future investigations.

A group of researchers has recently demonstrated that MLKL is a downstream target of RIP3 that binds to the cell membrane, leading to the pore formation and plasma membrane rupture following necroptotic signaling [27, 28]. However, it seems that MLKL was not involved in FOLE-induced necroptosis in IEC-6 cells, since pretreatment with NSA did not alleviate FOLE-induced cell death. On the contrary, it further enhanced the pro-necroptotic effect of FOLE when MLKL was inhibited (Fig. 4a). In addition to MLKL, high levels of activated RIP3 may cause aberrant energy metabolism and switch apoptosis to necrosis in some cell systems by stimulating the activity of metabolic enzymes including glycogen phosphorylase (PYGL), glutamate-ammonia ligase (GLUL), glutamate dehydrogenase 1 (GLUD1), fructose-1,6-bisphosphatase 2 (FBP2), fumarate hydratase (FH), glycosyltransferase 25 domain containing 1 (GLT25D1), and isocitrate dehydrogenase 1 (IDH1), leading to the overproduction of mitochondrial reactive oxygen species (ROS) and subsequent ROS-associated necrosis [29]. In this study, the results of ROS suggested that ROS overproduction instead of MLKL is probably responsible for the function of RIP3 in mediating FOLE-induced necroptosis. The results of reduced ROS production by Nec-1 suggested that ROS overproduction is a key downstream component of RIP1 activation following FOLE treatment; the results of enhanced ROS production by NSA suggested the possibility that even both MLKL and ROS are independent components downstream RIP3, ROS-mediated necrotic pathway may be enhanced when MLKL is inhibited. From this point of view, it seems that antioxidant vitamins may be one of the potential therapeutic strategies in preventing ROS overproduction and oxidative damage in intestinal mucosa. Currently, vitamin E is regarded as the most important lipid-soluble antioxidant in PN that helps to prevent membrane destabilizing lipid peroxidation by breaking lipid radical chain reactions [30]. As an important therapeutic strategy against PNALD, recent large, randomized, controlled trials have shown that vitamin E treatment results in significant improvement in steatosis, inflammation, ballooning, and resolution of steatohepatitis in adults without diabetes or cirrhosis [31–33], and the mechanisms against PNALD have been primarily attributed to the cellular antioxidant functions as well as prevention or reduction of oxidative stress in hepatocytes. However, it remains unclear whether supplementary vitamin E would also be able to attenuate intestinal oxidative stress like attenuating hepatic oxidative stress, as studies addressing the impact of PN on intestinal epithelium are limited currently. Therefore, it would be of great significance to address this issue in future studies. On the other hand, in addition to vitamin E, in the last years great efforts have been made in searching for nutrients and/or molecules able to favourably limit the oxidative damage under various physiological or pathological conditions. For example, carotenoids are currently considered beneficial substances, and among 50 carotenoids which are commonly employed in the human diet, 7 carotenoids (*β*-carotene, *α*-carotene, *β*-cryptoxanthin, lycopene, lutein, zeaxanthin and astaxanthin) have been widely studied on their benefits against the progress of cardiovascular disease owing to antioxidant and anti-inflammatory properties [34]. In some extent, more attention should be paid on the findings of novel antioxidants in the practice of PN administration because this may have important clinical significance when patients receiving prolonged PN present a higher antioxidant requirement for defense against oxidative stress (eg, chronic inflammation, sepsis and organ failure).

## Conclusion

In conclusion, this study demonstrated that FOLE, rather than SOLE or OOLE, induces programmed necrotic death (necroptosis) in IEC-6 cells, which requires RIP1/3 and is licensed by caspase 8. Our findings provided novel insights into the effect of FOLE on the homeostasis of intestinal epithelium in vitro, but further studies are needed to validate them in vivo.

## Additional file

Additional file 1: IEC-6 cells treated with FOLE. (AVI 1126 kb)

## Abbreviations

FOLE: Fish oil-derived lipid emulsion
LE: Lipid emulsion
MLKL: Mixed-lineage kinase domain-like protien
Nec-1: Necrostatin-1
OOLE: Olive oil-supplemented lipid emulsion
PN: Parenteral nutrition
PNALD: Parenteral nutrition associated liver disease
RIP: Receptor-interacting protein
ROS: Reactive oxygen species
SOLE: Soybean oil-based lipid emulsion
